# Characterizing the Metabolic and Immune Landscape of Non-small Cell Lung Cancer Reveals Prognostic Biomarkers Through Omics Data Integration

**DOI:** 10.3389/fcell.2021.702112

**Published:** 2021-07-06

**Authors:** Fengjiao Wang, Yuanfu Zhang, Yangyang Hao, Xuexin Li, Yue Qi, Mengyu Xin, Qifan Xiao, Peng Wang

**Affiliations:** ^1^Department of Thoracic Surgery, Harbin Medical University Cancer Hospital, Harbin, China; ^2^College of Bioinformatics Science and Technology, Harbin Medical University, Harbin, China; ^3^Department of Urinary Surgery, Harbin Medical University Cancer Hospital, Harbin, China

**Keywords:** omics data integration, single cell sequencing, prognostic biomarkers, NSCLC, cellular phenotypes

## Abstract

Non-small cell lung cancer (NSCLC) is one of the most common malignancies worldwide. The development of high-throughput single-cell RNA-sequencing (RNA-seq) technology and the advent of multi-omics have provided a solid basis for a systematic understanding of the heterogeneity in cancers. Although numerous studies have revealed the molecular features of NSCLC, it is important to identify and validate the molecular biomarkers related to specific NSCLC phenotypes at single-cell resolution. In this study, we analyzed and validated single-cell RNA-seq data by integrating multi-level omics data to identify key metabolic features and prognostic biomarkers in NSCLC. High-throughput single-cell RNA-seq data, including 4887 cellular gene expression profiles from NSCLC tissues, were analyzed. After pre-processing, the cells were clustered into 12 clusters using the t-SNE clustering algorithm, and the cell types were defined according to the marker genes. Malignant epithelial cells exhibit individual differences in molecular features and intra-tissue metabolic heterogeneity. We found that oxidative phosphorylation (OXPHOS) and glycolytic pathway activity are major contributors to intra-tissue metabolic heterogeneity of malignant epithelial cells and T cells. Furthermore, we constructed T-cell differentiation trajectories and identified several key genes that regulate the cellular phenotype. By screening for genes associated with T-cell differentiation using the Lasso algorithm and Cox risk regression, we identified four prognostic marker genes for NSCLC. In summary, our study revealed metabolic features and prognostic markers of NSCLC at single-cell resolution, which provides novel findings on molecular biomarkers and signatures of cancers.

## Introduction

Lung cancer is the leading cause of cancer-related deaths worldwide (Torre et al., 2015). Non-small cell lung cancer (NSCLC) and small cell lung cancer (SCLC) are the two classic histological subtypes of lung cancer. SCLC generally occurs in people of advanced age with a history of heavy smoking and accounts for approximately 15% of lung cancer cases (van Meerbeeck et al., 2011). NSCLC represents the remaining 85% of lung cancers and contains two main histological subtypes: lung adenocarcinoma (LUAD) and lung squamous cell carcinoma (LUSC) (Goldstraw et al., 2011). Since NSCLC has a wide range of genomic variation (Cancer Genome Atlas Research Network, 2014), it can respond better to immune checkpoint blockade (Rizvi et al., 2015), although there are exceptions (Topalian et al., 2016). With the development of single-cell sequencing technology, there is an increasing number of studies on identifying molecular features in the tumor microenvironment (TME) of NSCLC (Guo et al., 2018; Kim et al., 2020). However, there has been no significant improvement in the treatment of NSCLC. Therefore, it is necessary to explore the molecular features and prognostic markers of malignant cells at single-cell resolution.

During tumor tissue deterioration, cancer cells are reprogrammed by physiological mechanisms, including metabolic reprogramming, to support the demand for energy, biomass, and cellular communication (DeBerardinis and Chandel, 2016). Cell metabolism is influenced by genetic and environmental factors, including mutations that determine the direction of cell evolution, nutrients, and tissue origin (Boroughs and DeBerardinis, 2015; Pavlova and Thompson, 2016). The metabolic activity of cells is determined by the concentration of metabolically relevant molecules and biomolecule conversion rate, but these indicators are difficult to measure. Therefore, it is necessary to evaluate the expression of metabolic genes to indirectly determine the metabolic activity of cells (Puram et al., 2017).

The tumor tissue microenvironment (TME) is composed of malignant cells, fibroblasts, immune cells, and many other stromal cells (Bian et al., 2019). Each cell type plays an active role in tumor proliferation and metastasis. For example, cancer-associated fibroblasts (CAFs) assist in the invasion of tumor cells (Belle and DeNardo, 2019). Due to the different functions of each cell type, they all have specific metabolic requirements. Not only does each cell type have specific metabolic activities, but at the cellular level, each cell also has specific metabolic activities depending on its environment and evolutionary direction (Sottoriva et al., 2015; Reina-Campos et al., 2017; Reznik et al., 2018). Most conclusions about the metabolic features of the TME are derived from *in vitro* experiments (Carmona-Fontaine et al., 2017; Liu et al., 2018) or univariate measurements of metabolic enzyme expression (Miller et al., 2017), but these studies modify the TME to some extent.

The lymphocyte lineage is an important component of the TME, which has become a popular area in cancer immunotherapy. Cancer immunotherapy shows individual differences in the treatment of NSCLC (Topalian et al., 2012; Hellmann et al., 2019), which depends, in part, on the amount and properties of tumor-infiltrating lymphocytes (Rizvi et al., 2015; Huang et al., 2017). CD8+ and CD4+ T cells, as two subtypes of T cells, play an important role in the anti-tumor process. Although studies have pointed out a positive correlation between elevated CD8+ T cells and a good prognosis of cancer (Maimela et al., 2019), the mechanism that drives T-cell differentiation is unclear.

In this study, we explored the expression profiles of 4887 cells from the tumor tissues of four patients with NSCLC to identify the metabolic features of malignant cells and mechanisms that drive T-cell differentiation at the single-cell level, as well as to discover new therapeutic targets and prognostic markers.

## Materials and Methods

### Data Collection and Pre-processing

Single-cell RNA-seq profiles for NSCLC were collected from the Gene Expression Omnibus [GEO (Barrett et al., 2013);^1^] under accession number GSE117570. The profiles were derived from four tumor and four paracancerous tissue samples from four patients with NSCLC. To reveal the metabolic and immune features of NSCLC and identify therapeutic targets, four tumor samples containing 4,887 cells were used in this study. Fifteen low-quality cells were filtered out through quality control, leaving 4,872 cells. A gene was selected if it was expressed in at least 3 cells. The ineligible genes were filtered out, and 10,050 genes were retained for analysis. Considering the sequencing depth of these tumor samples, the Scran algorithm (Lun et al., 2016) was used to standardize the count data. The gene sets of the metabolic pathway were downloaded from the Molecular Signatures Database [MSigDB (Liberzon et al., 2011),^2^)]. Bulk RNA-seq profiles and clinical data of LUAD and LUSC belonging to NSCLC were collected from The Cancer Genome Atlas (Angelin et al., 2017) (TCGA,^3^). Moreover, the microarray sequencing data and survival data of the GSE3141 and GSE42127 datasets were obtained from the GEO database for the verification of prognostic markers. Information on all the samples used in this study is given in Supplementary Table 1.

### Dimensionality Reduction and Clustering of Cells

The preprocessed gene expression matrix and cell annotation information were encapsulated using the R package Seurat (version 3.2.2) (Satija et al., 2015). The top 3000 variant genes calculated by the standard deviation (SD) algorithm were used for principal component analysis (PCA). We adopt the strategy of using the least number of principal components to explain more data information (Butler et al., 2018). Thus, the top 16 principal components were manually selected for cell clustering analysis using the t-SNE algorithm. Marker genes of specific cell types in NSCLC tissues collected from published literature (Song et al., 2019) and CellMarker (Zhang et al., 2019b)^4^ database were used to define cell clusters.

### Metabolic Reprogramming Analysis of Malignant Cells

Due to the different origins and environments of malignant cells, specific malignant cell clusters may have unique metabolic mechanisms. The weighted relative pathway activity algorithm (Xiao et al., 2019) was used to evaluate specific metabolic features among specific malignant cell clusters. In this algorithm, the relative expression level of metabolic genes in each cell cluster was defined as the ratio of the mean expression value of cells in a specific cluster to the mean expression value of all cells. Furthermore, the activity score of a pathway of specific cell types was the weighted average of the relative expression of all genes in this pathway. Most importantly, weighting factors, the reciprocal of the number of pathways that include a certain gene, were used to eliminate commonalities between various metabolic pathways. Genes with low expression levels or high deletion rates in the pathway were also deleted to avoid the pathway activity score being affected by these factors. Cell type labels were randomly swapped 5000 times to construct a null distribution of pathway activity scores, which were used to examine the statistical significance of metabolic pathway activity scores in each cell cluster. For each pathway score, a *p*-value was calculated to assess whether the activity of the pathway was significantly higher or lower than the average.

### Evaluation of Intra-Tissue Metabolism Heterogeneity

The SD of each metabolic gene expression in malignant cell expression profiles, which reflects the variability of each metabolic gene in malignant cells, was calculated. Furthermore, genes were sorted in descending order according to their SD, and gene set enrichment analysis (GSEA) (Subramanian et al., 2005) was used to identify metabolic pathways enriched in metabolic genes with the highest variability using the R package fgsea (version 1.14.0).

### Evaluation of Developmental Trajectory of T Cells

Based on the definition of various T-cell characteristics in previous studies (O’Shea and Paul, 2010; Michalek et al., 2011), CD4+ (Th/Treg) T cells and CD8+ T cells were distinguished from the original T-cell clusters. For these T-cell types, pseudo-time developmental trajectories were constructed by monocle (Qiu et al., 2017) (version: 2.16.0), which is an algorithm that describes multiple fate decisions in a completely unsupervised manner using the reverse graph embedding method. Multiple branches and nodes were observed throughout the developmental trajectory, and cells on the same branch were considered to have the same state.

### Construction of Transcriptional Regulatory Network

The count of biomolecules varies during T-cell development. Pearson correlation analysis was used to explore genes related to the development of T cells. The correlation coefficients of genes with the developmental processes of CD4+ and CD8+ T cells was evaluated. Genes with Pearson’s correlation coefficient | R| > 0.2 (Shin et al., 2015) and *p*-value < 0.05, were defined as genes associated with T-cell development, including those associated with the development of CD4+ and CD8+ T cells. To explore the effects of these genes on the development of T cells and their transcriptional regulatory relationships, functional enrichment analysis was conducted, and a transcriptional regulatory network was constructed. The R package clusterProfiler (version 3.16.1) (Yu et al., 2012) was used to examine the enrichment of genes positive related to T-cell development in Gene Ontology (GO) (The Gene Ontology Consortium, 2019) function nodes and Kyoto Encyclopedia of Genes and Genomes (KEGG) (Kanehisa et al., 2017) pathways. Human transcription factor data were collected from the AnimalTFDB (Hu et al., 2019) 3.0 database^5^. Data for the relationship between transcription factors and their target gene regulation were collected from TRRUST (Han et al., 2018)^6^ and ORTI (Vafaee et al., 2016)^7^ databases.

### Construction of Survival Prediction Model Based on Critical Factors

The lasso algorithm was used to screen for critical genes from the identified T-cell development-related genes, which are associated with the overall survival (OS) of patients with LUAD. Based on these critical factors, a multivariate Cox regression model was constructed, and the significance indicator for each gene was calculated. Furthermore, to predict the OS of patients with LUSC, the critical genes with a *p-*value < 0.05 were retained to establish a risk prediction model and nomogram for survival analysis. The reliability of this risk prediction model was depicted by the receiver operating characteristic (ROC) curve, and the area under the curve (AUC) was also calculated. Furthermore, the prognostic markers and regression coefficients obtained from the multivariate Cox regression model were used to construct a risk score model. The samples were divided into high-risk and low-risk groups based on the median risk score calculated by the risk score model, and Kaplan-Meier survival analysis was performed to study the difference in OS between these two groups using the bilateral logarithmic rank test. These prognostic marker genes used in the risk score model of LUAD were also connected to other NSCLC survival data.

## Results

### Individual Differences in Malignant Transformation of Tumor

**FIGURE 1:**
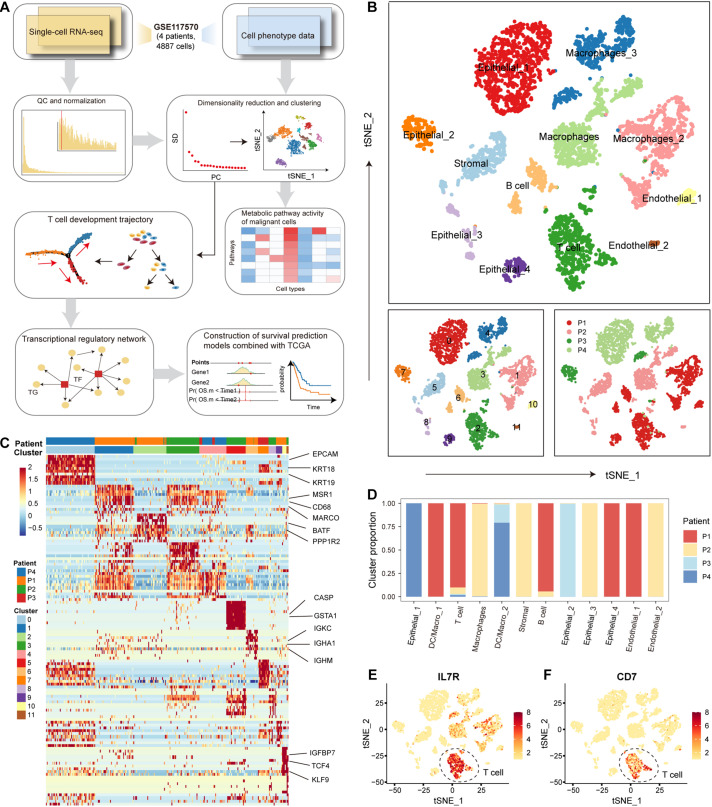
The cellular landscape of non-small cell lung cancer (NSCLC). **(A)** Workflow diagram representing the pipeline for scRNA-seq data analysis. **(B)** Tumor cells were clustered by the t-SNE clustering algorithm into 12 clusters with a specific color marker. **(C)** Heatmap of differentially expressed genes in each cluster. Expression levels of the top 30 genes (rows) that are differentially expressed in each cluster (column). Marker genes in each cluster were annotated. **(D)** Bar plot of the origin of the cells in each cell type. The horizontal axis is the 12 cell types, and the vertical axis is the proportion of cells. **(E)** Distribution of *IL7* gene expression in all tumor cells. The higher the gene expression, the darker the color. **(F)** Same as in **(E)** but for the distribution of gene *CD7*.

We used a calculational pipeline to analyze the gene expression profile of NSCLC at the single-cell level (Figure 1A). After quality control and normalization, gene expression profiles of 4872 cells from tumors of 4 patients with NSCLC were used for subsequent analysis. All cells were divided into 12 clusters according to the t-SNE clustering algorithm (Figure 1B). We annotated the cell types of the 12 cell clusters spanning malignant epithelial cells (0, 7, 8, and 9 clusters marked by EPCAM and KRT18/19), macrophages (1, 3, and 4 clusters marked by MSR1, CD68, and MARCO), T cells (2 clusters marked by CD7, BATF, PPP1R2, and PPP2R5C), stromal cells (5 clusters marked by CASP and GSTA1), B cells (6 clusters marked by IGKC, IGHA1, IGHG1, and IGHM), endothelial cells (10 and 11 clusters marked by IGFBP7, TCF4, KLF9, and ITGB1) (Figures 1B,C). We found 4 clusters of malignant epithelial cells corresponding to their tumors of origin (i.e., from which tumor the cell was derived) and 8 clusters of other stromal cells also corresponding to their tumors of origin, suggesting that NSCLC tissues have obvious individual differences (Figures 1B,D). The first patient (patient_1) had a significantly higher proportion of T and B cells compared to the other three patients (Figure 1D), suggesting that patient_1 may be more suitable for immune-targeted therapy. The results of the following studies support our inference. We found significantly high expression of *IL7R* and *CD7* genes in T cells (Figures 1E,F). Signaling pathways mediated by IL7R are critical for T-cell development and homeostasis *in vivo*, and aberrant IL7R activation was strongly associated with the development of human T-cell leukemogenesis (Zenatti et al., 2011; Yasunaga, 2020). Blocking the expression of CD7, an immune transmembrane protein, is beneficial in the treatment of T-cell malignancies (Gomes-Silva et al., 2017; Png et al., 2017). These findings suggest that individual differences in NSCLC uncovered at single-cell resolution are critical for the development of precision medicine.

### Intra-Tissue Metabolic Heterogeneity of Malignant Cells

**FIGURE 2:**
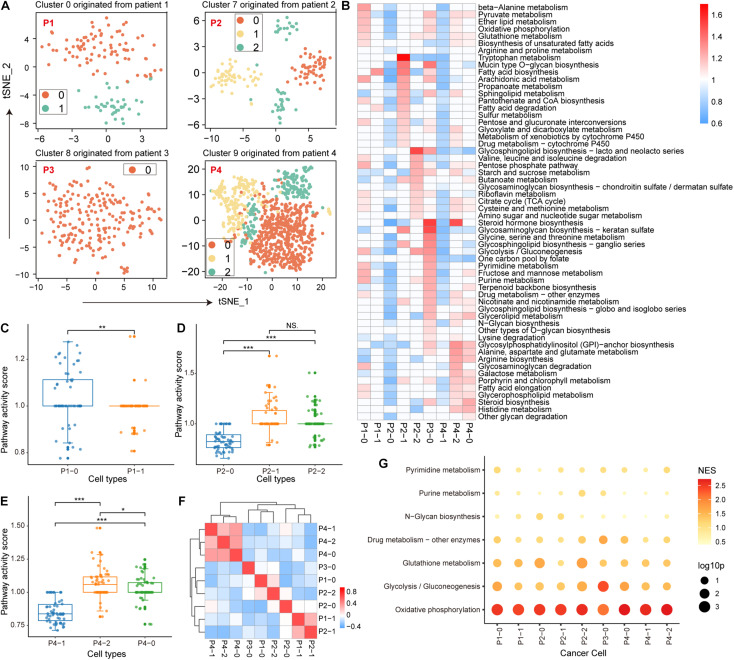
Metabolic features of malignant epithelial cells. **(A)** Four clusters of malignant epithelial cells originating from four patients with non-small cell lung cancer (NSCLC) were individually re-clustered using t-SNE. **(B)** Metabolic pathway activity in each cell cluster. Values with statistically non-significant pathway activity (random permutation test, *p* > 0.05) were shown as blank. **(C–E)** Boxplot of pathway activity scores of malignant cell clusters originating from patients 1, 2, and 4. **(F)** Correlation of each malignant cell cluster in metabolic pathway activity scores. Additionally, ^∗^*p* < 0.05, ^∗∗^*p* < 0.01, ^∗∗∗^*p* < 0.001. **(G)** Enrichment results of metabolic pathways in variant genes using gene set enrichment analysis (GSEA). The size of the bubbles represents statistical significance and the shade of the color represents the normalized enrichment score (NES).

During the malignant transformation of the tumor, the metabolic processes of each cell are influenced by the microenvironment, including nutrient concentrations and interactions with other cells in the same space. Therefore, it is intriguing to investigate the metabolic features of malignant cell clusters and the microenvironmental factors that contribute to the intra-tissue metabolic differences. For malignant epithelial cell clusters from four patients, we re-clustered the cells according to their metabolic gene expression profiles. The original 4 clusters of malignant cells were re-clustered into 9 clusters (Supplementary Figure 1) 2 clusters from patient 1, 3 clusters from patient 2, 1 cluster from patient 3, and 3 clusters from patient 4. We also separately re-clustered epithelial cells based on the origin of tumor tissue; the cells were clustered into 9 clusters that were similar to the above clustering results (Figure 2A), indicating the individual differences of malignant epithelial cells and the stability of the subtype. Furthermore, the weighted pathway activity score algorithm was used to measure the relative activity of the metabolic pathways of these 9 clusters. Among the 85 metabolic pathways, 58 pathways containing at least 5 genes had significantly upregulated activity scores (pathway activity score >1 and permutation test *p*-value < 0.01) in at least one cell cluster compared to other cell clusters (Figure 2B). Malignant cell clusters in patient 3 (p3_0) had the highest number of significantly upregulated metabolic pathways (36 pathways upregulated in p3_0 compared to 30 in p4_2, 22 in p4_0, 21 in p1_0, and <10 in other clusters; Supplementary Table 2), which included many different parts of cellular metabolism, such as glycolysis, oxidative phosphorylation (OXPHOS), and the pentose phosphate pathway (Figure 2B). We found significant differences between the metabolic pathway activity scores of malignant cell clusters in patients 1–3 (Figures 2C–E), suggesting that the activity of metabolic pathways is determined by tumor origin. Although patient 3, cluster 2 of patient 4, and cluster 0 of patient 1 had more upregulated metabolic pathways compared with other clusters, their activity scores demonstrated a poor correlation (Figure 2F), indicating specific metabolic reprogramming between tumors.

Next, we identified the microenvironmental factors that contribute to the intra-tissue metabolic heterogeneity. We performed GSEA to identify metabolic pathways enriched in genes explaining most of the variation among the malignant cells of each tumor cluster. We found that OXPHOS was the top-scoring pathway in most tumor clusters (Figure 2G). Similarly, glycolysis also made a major contribution to the metabolic heterogeneity of several tumor clusters, indicating that energy metabolic factors (mitochondrial activity) are important contributors to intra-tissue metabolic heterogeneity.

### Metabolic Features During T-Cell Differentiation

**FIGURE 3:**
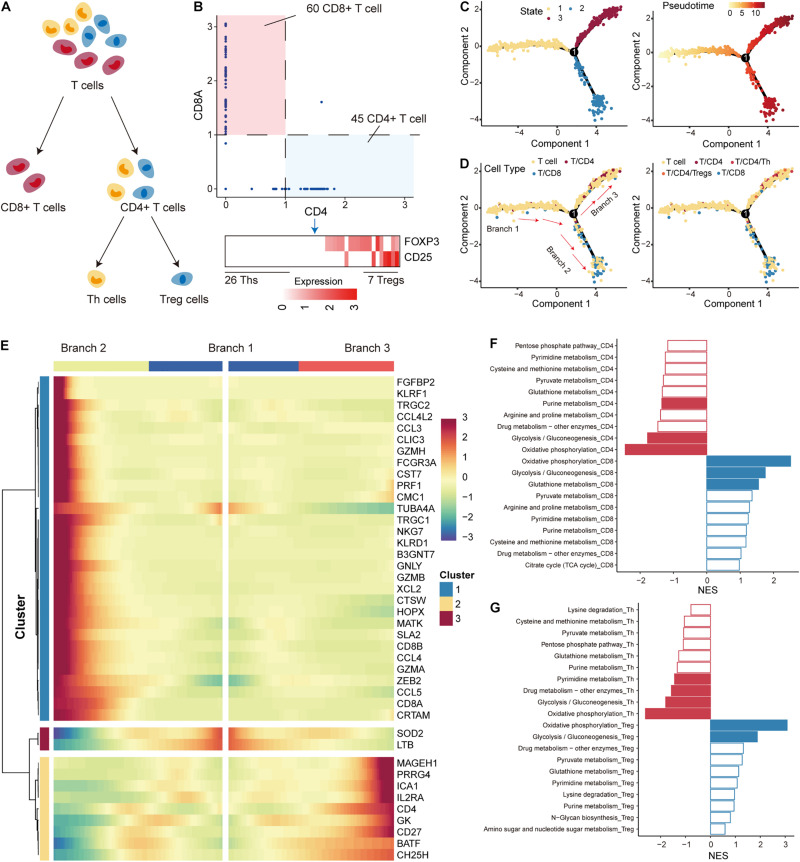
Differentiation trajectory and metabolic features of T cells. **(A)** The panel shows the differentiation process of T cells; T cells were divided into CD4+ T, CD8+ T, Th, and Treg cells. **(B)** Expression levels of marker genes (including CD4, CD8A, FOXP3, and CD25) were used to separate T-cell subtypes in non-small cell lung cancer (NSCLC). **(C)** The pseudo-time trajectory of T-cell differentiation. Each point represents a cell and is marked with the cell state (left) and pseudo-time (right). **(D)** Same as in **(C)** but for cells marked with T-cell subtypes. **(E)** The heatmap shows the branch-dependent genes at branch point 1. The center of the heatmap is branch B1, the left is B2, and the right is B3. **(F)** Top 10 metabolic pathways enriched in CD4+ or CD8+ T cells. Significantly enriched pathways with gene set enrichment analysis (GSEA) *p*-values < 0.05 are highlighted in red (CD4) or blue (CD8). **(G)** Top 10 metabolic pathways enriched in Th or Treg cells. Significantly enriched pathways with GSEA *p*-values < 0.05 are highlighted in red (Th) or blue (Treg).

In the TME, immune cells that differentiate into subtypes with distinct roles constitute an important component. Next, we used single-cell RNA-seq profile to characterize the developmental trajectory and metabolic features of T cells, which constitute the major immune cell population. According to previous studies on T cells, T cells were first separated into CD4+ and CD8+ subtypes based on the expression of the cell-surface markers CD4 and CD8A (O’Shea and Paul, 2010) (Figure 3A). CD4+ T cells were further differentiated into regulatory T cells (Tregs) and T helper cells (Ths) based on the expression of FOXP3 and CD25 (Michalek et al., 2011) (Figure 3A). We defined T cells with CD8A expression less than 1 and CD4 expression more than 1 as CD4+ T cells (45 CD4+ T cells), and cells with the opposite expression status were defined as CD8+ T cells (60 CD8+ T cells; Figure 3B; Supplementary Figure 2). Furthermore, CD4+ T cells with a sum of FOXP3 and CD25 expression higher than 2 were identified as Tregs (7 Tregs), and those cells that did not express FOXP3 and CD25 were defined as Ths (26 Ths; Figure 3B; Supplementary Figure 2). The R package monocle was then used to emulate the pseudo-developmental trajectory of the T-cell subpopulations. Three branches (defined as “B1,” “B2,” and “B3”) were found in the developmental trajectory of T cells, which were divided into three states (Figure 3C). Based on the visualization results, CD4+ T cells, including Tregs and Ths, were mainly concentrated on state 2 and CD8+ T cells were mainly concentrated on state 3 (Figure 3D). Based on the above information, we concluded that the cell development from B1 through branch point 1 to state 3 correspond to the conversion of T cells to CD4+ T cells, and the cell development from B1 through branch point 1 to state 2 correspond to the conversion of T cells to CD8+ T cells. We identified multiple branch-dependent genes for branching point 1 (*p* < 0.01), which were closely associated with T-cell differentiation (Figure 3E).

T-cell differentiation was accompanied by reprogramming of metabolic pathways to satisfy the physiological demands of the new cellular state (DeBerardinis and Chandel, 2016; Xiao et al., 2019). We then performed GSEA analysis to identify metabolic pathways enriched in highly variable genes of each subtype. We found that OXPHOS and glycolysis had the top normalized functional enrichment scores (NESs) in CD4+ T and CD8+ T cells, suggesting that mitochondrial activity is also a major contributor to metabolic heterogeneity among T cells (Figure 3F). Glutathione metabolism and purine metabolism were found to be important metabolic pathways that distinguished the T-cell subtypes (Figure 3F). Interestingly, OXPHOS and glycolysis also had the highest NES in Tregs and Ths cells (Figure 3G). We found that the rates of glycolysis were upregulated in Tregs compared to Ths cells (Supplementary Figure 3), which seems to contradict previous studies showing that Ths are more susceptible to glycolysis than Tregs derived from healthy mice without tumors (Michalek et al., 2011). In contrast, the preference for OXPHOS in Tregs was consistent with previous studies (Ma et al., 2016; Angelin et al., 2017; Buck et al., 2017), highlighting that enhanced mitochondrial oxidative metabolism is a characteristic of Tregs. These results suggest that the metabolic features of T cells in the TME differ from those of normal tissues.

### Key Factors in T-Cell Differentiation Correlate With Tumor Metastasis and Patient Prognosis

**FIGURE 4:**
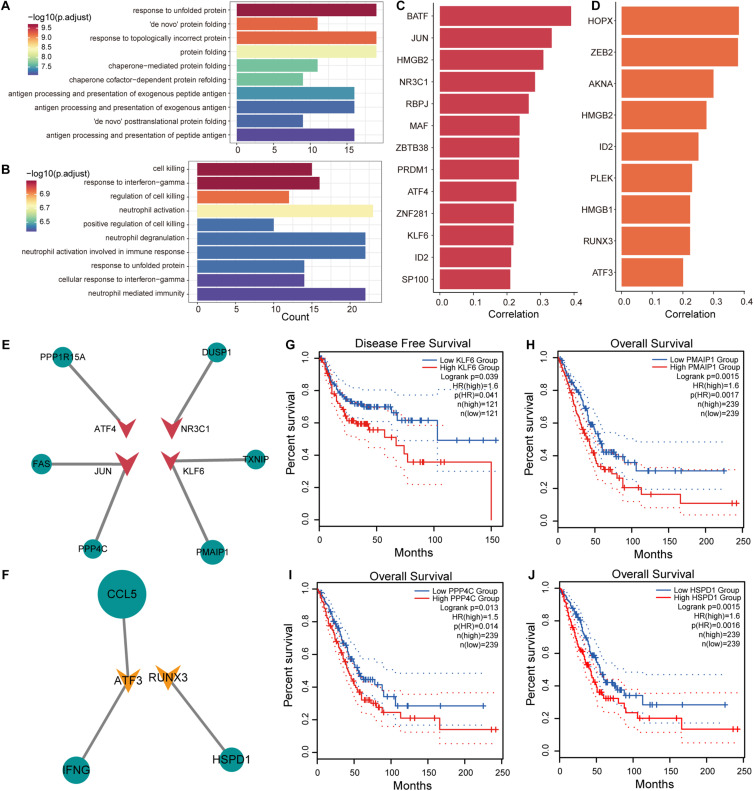
Transcriptional regulation of genes associated with T-cell differentiation. **(A)** Enrichment results of upregulated genes associated with T/CD4+ differentiation in the gene ontology (GO) term. **(B)** Same as in **(A)** but for genes associated with T/CD8+ differentiation. **(C)** Transcription factor (TF) lists selected from genes positively associated with T/CD4+ differentiation and their Pearson correlation coefficients with pseudo-time. **(D)** Same as in **(C)** but for TF lists selected from genes associated with T/CD8+ differentiation. **(E)** The transcriptional regulatory network of TFs and target genes in genes related to T/CD4+ differentiation. The size of the nodes in the network represents the correlation coefficient, the circle represents the target gene, and the triangle represents the TF. **(F)** Same as in **(E)** but for genes related to T/CD8+ differentiation. **(G)** Survival curves comparing disease-free survival (DFS) of patients with high and low *KLF6* gene expression. **(H–J)** Survival curves comparing overall survival (OS) of patients with high and low *PMAIP1*, *PPP4C*, and *HSPD1* gene expression.

T cells in tumor tissues have specific metabolic and physiological mechanisms that are influenced by the TME. To explore the effects of genes associated with T-cell differentiation on cellular physiological functions, Pearson correlation analysis was used to identify genes associated with the T-cell differentiation process. We identified 308 genes (216 positive and 92 negative related genes) and 284 genes (187 positive and 92 negative related genes) that were associated with T/CD4+ differentiation and T/CD8+ differentiation, respectively. The R package clusterprofiler was used for GO functional enrichment and KEGG pathway analysis of genes related to T-cell differentiation. We found that genes upregulated in T/CD4+ differentiation were significantly enriched in functional pathways associated with antigen processing (Figure 4A and Supplementary Figure 4A) and presentation, and genes upregulated in T/CD8+ differentiation were significantly enriched in the regulation of cell killing and the immune functions in which neutrophils are involved (Figure 4B and Supplementary Figure 4B). These findings are consistent with previous studies revealing the function of CD4+ and CD8+ T cells (Imanishi and Saito, 2020), which supports the reliability of cell cluster identification and T-cell differentiation trajectory identification in this study.

Identification of key factors that promote T-cell differentiation and the formation of specific metabolic mechanisms for T-cell is of substantial importance for the treatment of NSCLC. We focused on transcription factors (TFs) that regulate the expression efficiency of target genes. Using human TF data collected from AnimalTFDB (Hu et al., 2019), 13 and 9 TFs were identified from genes associated with T/CD4+ differentiation and T/CD8+ differentiation (Figures 4C,D). In combination with other genes associated with T-cell differentiation, we constructed transcriptional regulatory networks associated with T/CD4+ and T/CD8+ differentiation (Figures 4E,F), respectively. We found that high expression of TF KLF6 was associated with poor disease-free survival (DFS) in patients with NSCLC and that the high expression of its target gene, *PMAIP1*, was associated with poor OS (Figures 4G,H). Previous studies have confirmed that KLF6 is a key TF that participates in the activation of T cells (Palau et al., 2013), suggesting that KLF6 can be used as a potential target for regulating the differentiation of T cells and assisting in immunotherapy. The variation in KLF6 expression was also associated with metastatic potential and poor prognosis of patients with NSCLC (DiFeo et al., 2009; Zhang et al., 2019a). PPP4C is required for extracellular skeleton composition in cell metastasis (Martin-Granados et al., 2008), which was highly expressed in CD4+ T cells and was associated with poor OS (Figure 4I). High expression of HSPD1 in CD8+ T cells was strongly associated with poorer patient OS (Figure 4J). Moreover, high expression of HSPD1 was shown to inhibit E-cadherin expression to promote cell metastasis (Kang et al., 2019). The HSP60 protein encoded by *HSPD1* could be used for exosomal antigen presentation to dendritic cells (DCs) to inhibit the differentiation of CD4+ T cells (Cui et al., 2019), suggesting that inhibiting the differentiation of CD4+ T cells by regulating the expression of KLF6 can be used as a strategy to alleviate immunosuppression. Additionally, we have precisely defined the functions of target genes that are positively associated with CD4+ and CD8+ T-cell differentiation using Metascape (Zhou et al., 2019) (Supplementary Tables 3, 4). Taken together, these results suggest that the key genes that drive T-cell differentiation can stimulate tumor invasion and metastasis and could be used as potential therapeutic targets.

### Identifying Prognostic Markers for NSCLC in Combination With Public Data

**FIGURE 5:**
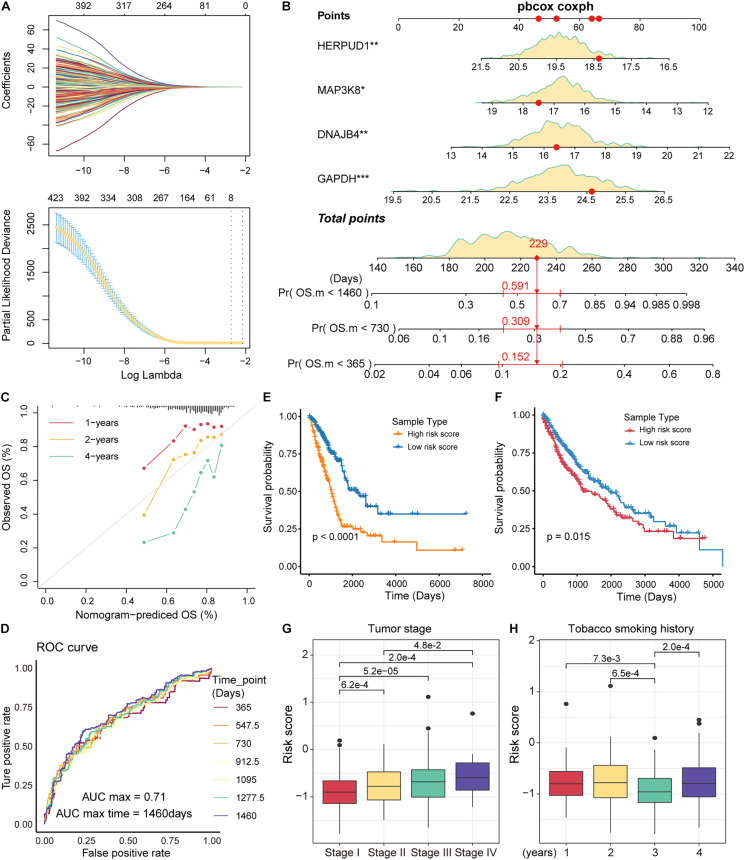
Identification of prognostic markers in non-small cell lung cancer (NSCLC). **(A)** The curves reflect the relationship between the regression coefficient and λ value. The features were selected by the lasso regression model. **(B)** Nomogram for survival risk prediction of 1, 2, and 4 years. The model contains four feature genes (prognostic markers). **(C)** Calibration curve of the nomogram. **(D)** Receiver operating characteristic (ROC) plots of the outcomes predicted by the risk regression model for seven time points. The different colored curves represent specific time points. **(E)** Kaplan-Meier (KM) curves for the survival time (days) of lung adenocarcinoma (LUAD) samples in high- and low-risk categories. The log-rank test was used to calculate statistical significance. **(F)** Same as in **(E)** but for the survival of lung squamous cell carcinoma (LUSC) samples. **(G)** Box plots of risk scores for different tumor stage samples. The rank-sum test was used to statistically measure differences between groups. **(H)** Same as in **(G)** but for samples with different smoking histories.

The immune microenvironment of tumors is closely related to tumor development (Lei et al., 2020). As an important component of the immune microenvironment, the dynamics of T cells at the molecular and cellular levels significantly affect tumor development and metastasis. It is intriguing to identify the markers associated with the prognosis of patients with NSCLC from genes related to T-cell differentiation. Next, we used lasso regression to screen for genes that are strongly associated with patient prognosis of LUAD, and 8 genes associated with patient prognosis were identified (Figure 5A). The multivariate Cox regression models were used to fit these eight feature genes, four of which, *HERPUD1*, *MAP3K8*, *GAPDH*, and *DNAJB4*, were significantly associated with the risk of death in patients (*p* < 0.05; Supplementary Figure 5). Nomograms were used to predict the probability of death at 1, 2, and 4 years (Figure 5B). The results of the calibration curve showed the strong stability of the risk prediction model (Figure 5C). To identify the best predictive time points for the risk prediction model, we split the 4-year period into six time periods and evaluated the prediction results using the ROC curve. We found that the risk prediction results reached a maximum AUC value of 0.71, in the fourth year (1460 days; Figure 5D). Furthermore, we used regression coefficients for HERPUD1, MAP3K8, GAPDH, and DNAJB4 to construct risk score models as follows: risk score = −0.197^∗^MAP3K8 −0.261^∗^HERPUD1+ 0.185^∗^GAPDH+ 0.191^∗^DNAJB4 and calculated the risk score for each LUAD tumor sample. The samples were divided into two categories (high-risk and low-risk) based on the median of risk scores, and we found that the high-risk samples were associated with poorer OS in LUAD (Figure 5E). The four prognostic markers, HERPUD1, MAP3K8, GAPDH, and DNAJB4, have also been used to predict survival in patients with LUSC. In LUSC, the high-risk samples also showed poorer OS (Figure 5F). Additionally, we combined the four prognostic markers identified and samples of NSCLC obtained from the GEO database to reconstruct the risk score model. We found that high-risk scores were significantly associated with poorer survival, which was similar to previous predictions (Supplementary Figure 6). These results suggest that HERPUD1, MAP3K8, GAPDH, and DNAJB4 can be used as prognostic markers in NSCLC. By combining clinical information from the LUAD sample with the risk score, we found that patients with stage IV cancer had a significantly higher risk score (Figure 5G). Although smoking could increase the risk of lung cancer (Bade and Dela Cruz, 2020), the history of smoking does not show a positive correlation with the prognostic risk (Figure 5H), indicating that specific tobacco tolerance is caused by individual genetic differences.

## Discussion

In this study, we used a computational pipeline to reveal the biological information contained in the single-cell RNA profiles of NSCLC. By clustering cells from the tumor tissues of four patients, individual differences in the metabolic landscapes of malignant epithelial cells were revealed. We characterized the metabolic characteristics of the malignant epithelial cells. GSEA revealed that OXPHOS and glycolysis are the major contributors to the intra-tissue metabolic heterogeneity of malignant cells. We constructed the differentiation trajectory of T cells using the monocle tool and revealed that T-cell subtypes have different metabolic features from normal tissues to adapt to the TME. In the process of T-cell differentiation, we found that the key genes that drive T-cell differentiation could serve as potential therapeutic targets. Finally, we constructed a survival risk prediction model and identified HERPUD1, MAP3K8, GAPDH, and DNAJB4 as prognostic markers for NSCLC.

The TME includes not only the tumor cells but also the surrounding fibroblasts, immune and inflammatory cells, glial cells, and other cells (Arneth, 2019; Hinshaw and Shevde, 2019). Tumor proliferation and metastasis are drove by malignant cells and other stromal cells. Therefore, we focused on malignant epithelial cells and T cells, which are important components of immune cells. Although numerous studies have explored the features of immune cells and signaling pathways in the NSCLC microenvironment at a single-cell resolution (Guo et al., 2018; Clarke et al., 2019; Maynard et al., 2020), few studies have delved into the metabolic programming of malignant cells and the driving mechanism of T-cell differentiation (Kim et al., 2020). In this study, we characterized the metabolic characteristics of malignant cells and T-cell subtypes. Considering that numerous genes appear repeatedly in multiple metabolic pathways, we did not use traditional gene set variation analysis (GAVA) (Hanzelmann et al., 2013) to calculate metabolic pathway activity. The weighted relative pathway activity algorithm can highlight the activity features of each pathway to avoid the effect of under- and over-expression of overlapping genes in multiple pathways.

Tumors are formed when normal cells continue to proliferate in an uncontrolled manner due to the loss of cell cycle control (Radomska et al., 2019). Tumors develop different directions of mutations and malignant evolution due to different cancer-causing agents. Therefore, we aimed to identify the individual differences in the metabolic activity of malignant epithelial cells. Malignant cells are influenced by their tissue environment and show significant heterogeneity in mitochondrial activity. The differentiation process of T cells is accompanied by metabolic reprogramming. We found that Tregs have higher glycolytic activity than Ths cells in tumor tissues, which is opposite to that in normal tissues. Tregs play a crucial role in maintaining immune tolerance, and their abnormal expression can lead to autoimmune diseases (Barbi et al., 2014). The reprogramming of energy metabolism in Treg cells may be an important contributor to immune dysfunction in tumor tissues. We found that genes related to T-cell differentiation can serve as prognostic markers for patients with NSCLC, which may be due to the remodeling of T-cell molecular mechanisms that affect tumor proliferation and metastasis.

In conclusion, this study describes the metabolic and immune landscapes of NSCLC at the single-cell level. Marker genes associated with patient prognosis were identified through the construction of survival risk models. Although we have only analyzed NSCLC in terms of metabolic pathways and T-cell differentiation, this study provides insights into tumor proliferation and prognosis of patients with NSCLC. The findings of this study may provide theoretical guidance for the diagnosis and treatment of NSCLC.

## Data Availability Statement

The datasets presented in this study can be found in online repositories. The names of the repository/repositories and accession number(s) can be found in the article/Supplementary Material.

## Author Contributions

PW, FW, and YZ conceived and designed the experiments. FW, YZ, and YH analyzed the data. XL and YQ collected the data. FW, MX, and QX validated the methods and data. FW and YZ wrote the manuscript. All authors have read and approved the final version of the manuscript.

## Conflict of Interest

The authors declare that the research was conducted in the absence of any commercial or financial relationships that could be construed as a potential conflict of interest.

## Footnotes

1 https://www.ncbi.nlm.nih.gov/gds

2 http://www.broadinstitute.org/msigdb

3 https://portal.gdc.cancer.gov/

4 http://biocc.hrbmu.edu.cn/CellMarker/

5 http://bioinfo.life.hust.edu.cn/AnimalTFDB/

6 https://www.grnpedia.org/trrust/

7 http://orti.sydney.edu.au/about.html
