# Supplementary material for: Characterizing the Metabolic and Immune Landscape of Non-small Cell Lung Cancer Reveals Prognostic Biomarkers Through Omics Data Integration
Source: Front Cell Dev Biol. 2021 Jul 6;9:702112. doi: 10.3389/fcell.2021.702112 (PMC8290418; doi:10.3389/fcell.2021.702112)
Supplement: Supplementary file 1 [file Data_Sheet_1.docx]

**Supplementary Figures**

**
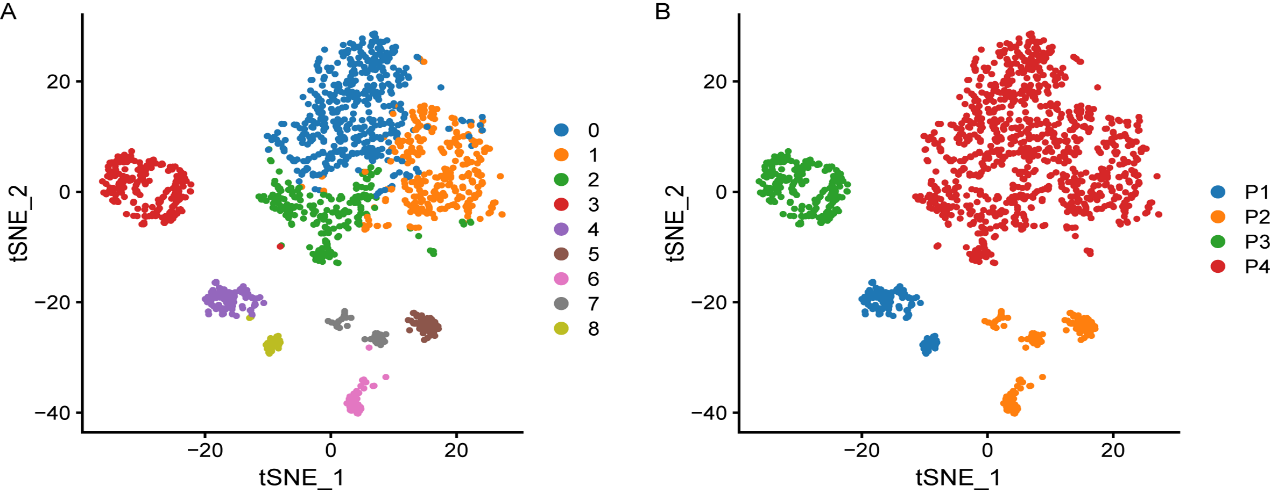
**

**Figure S1. (Related to Figure 2) (A)** Malignant epithelial cells from four patients were clustered by the t-SNE algorithm into 9 clusters with a specific color marker. **(B)** Same as in **(A)** but epithelial cells were marked by tumor tissue origin.

**
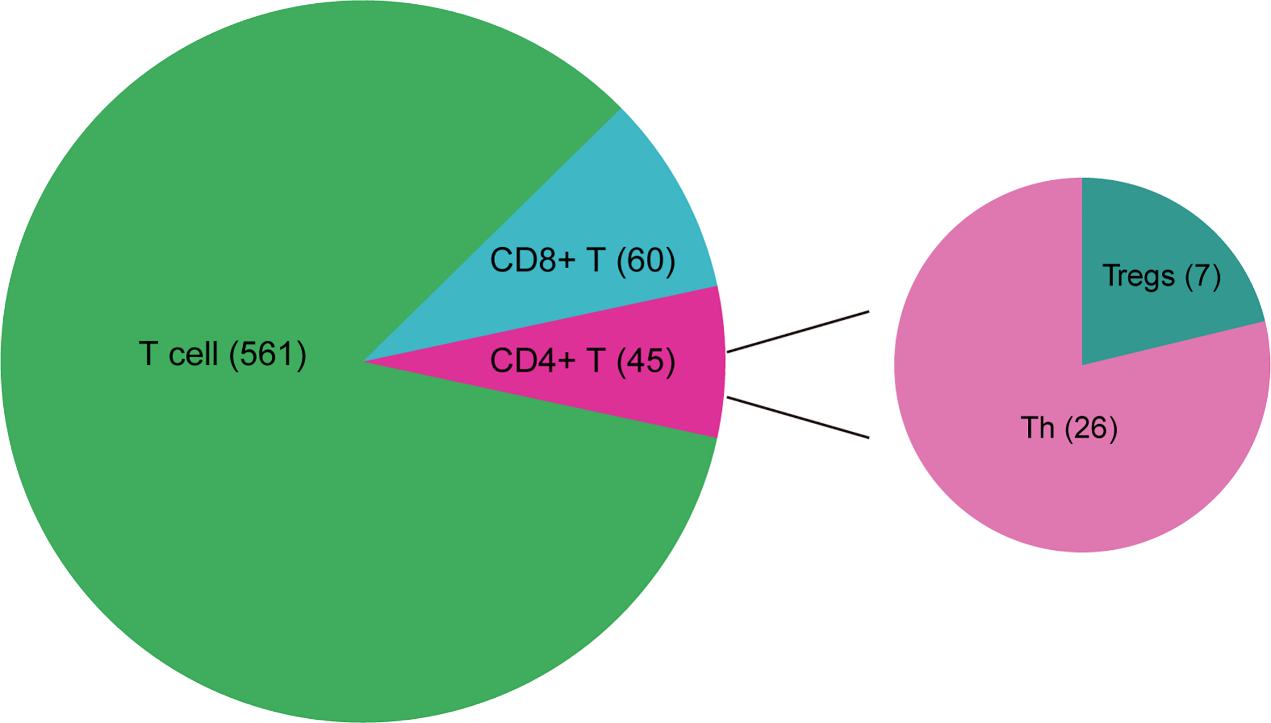
**

**Figure S2. (Related to Figure 3)** Pie chart for visualizing cell proportions. Left panel: the number of T cells, CD8+ T cells and CD4+ T cells. Right panel: the number of Th and Treg cells.


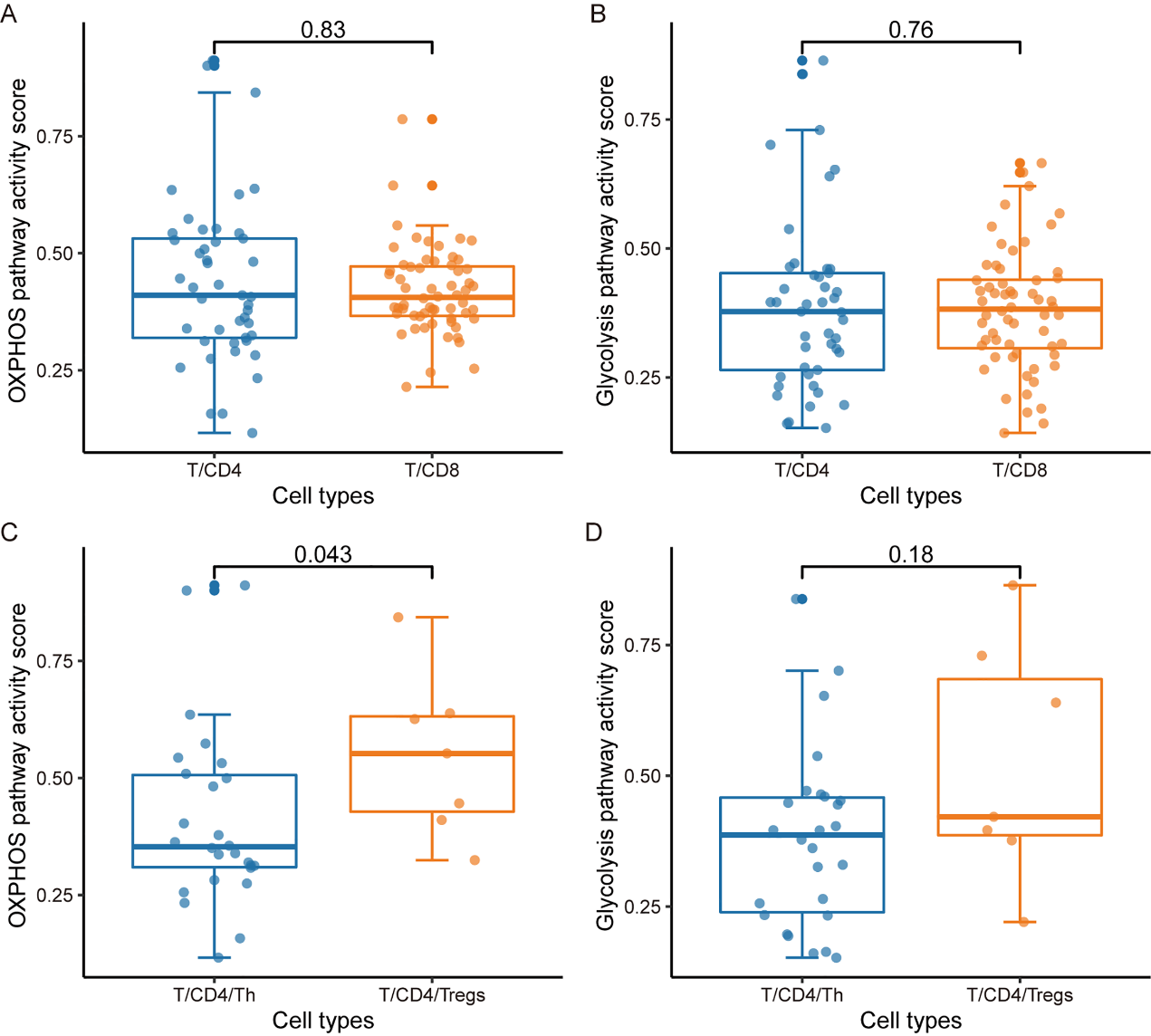


**Figure S3. (Related to Figure 3) (A)** Box plots of OXPHOS pathway activity scores for CD4+ and CD8+ T cells. The rank-sum test was used to statistically measure differences between groups. **(B)** Box plots of glycolysis pathway activity scores for CD4+ and CD8+ T cells. **(C)** Same as in **(A)** but for Th and Treg cells. **(D)** Same as in **(B)** but for Th and Treg cells.

**
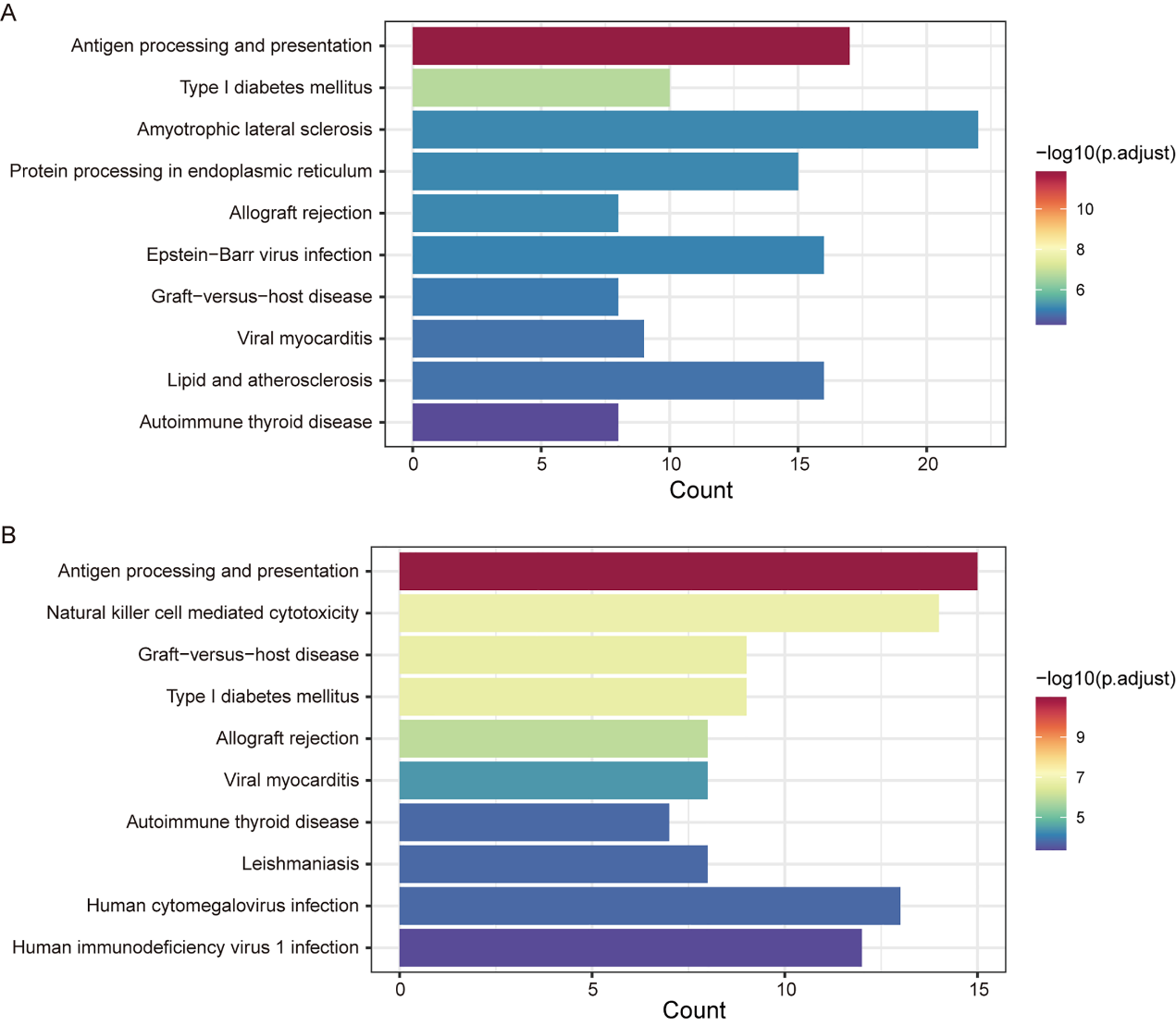
**

**Figure S4. (Related to Figure 4) (A)** Enrichment results of up-regulated genes associated with T/CD4+ differentiation in the KEGG pathway. **(B)** Same as in **(A)** but for genes associated with T/CD8+ differentiation.

**
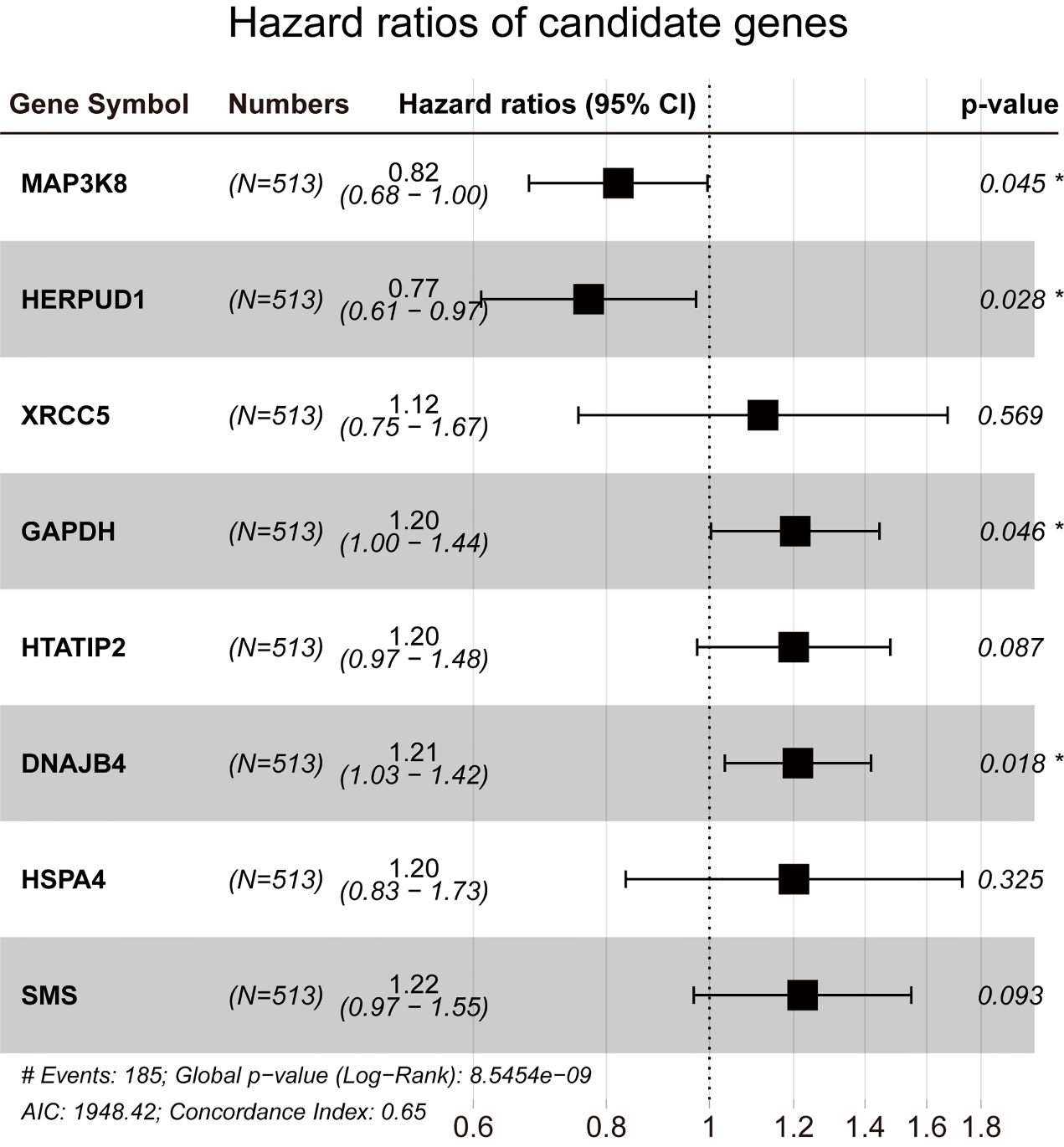
**

**Figure S5. (Related to Figure 5)** Forest plots for the multivariate Cox risk regression models.


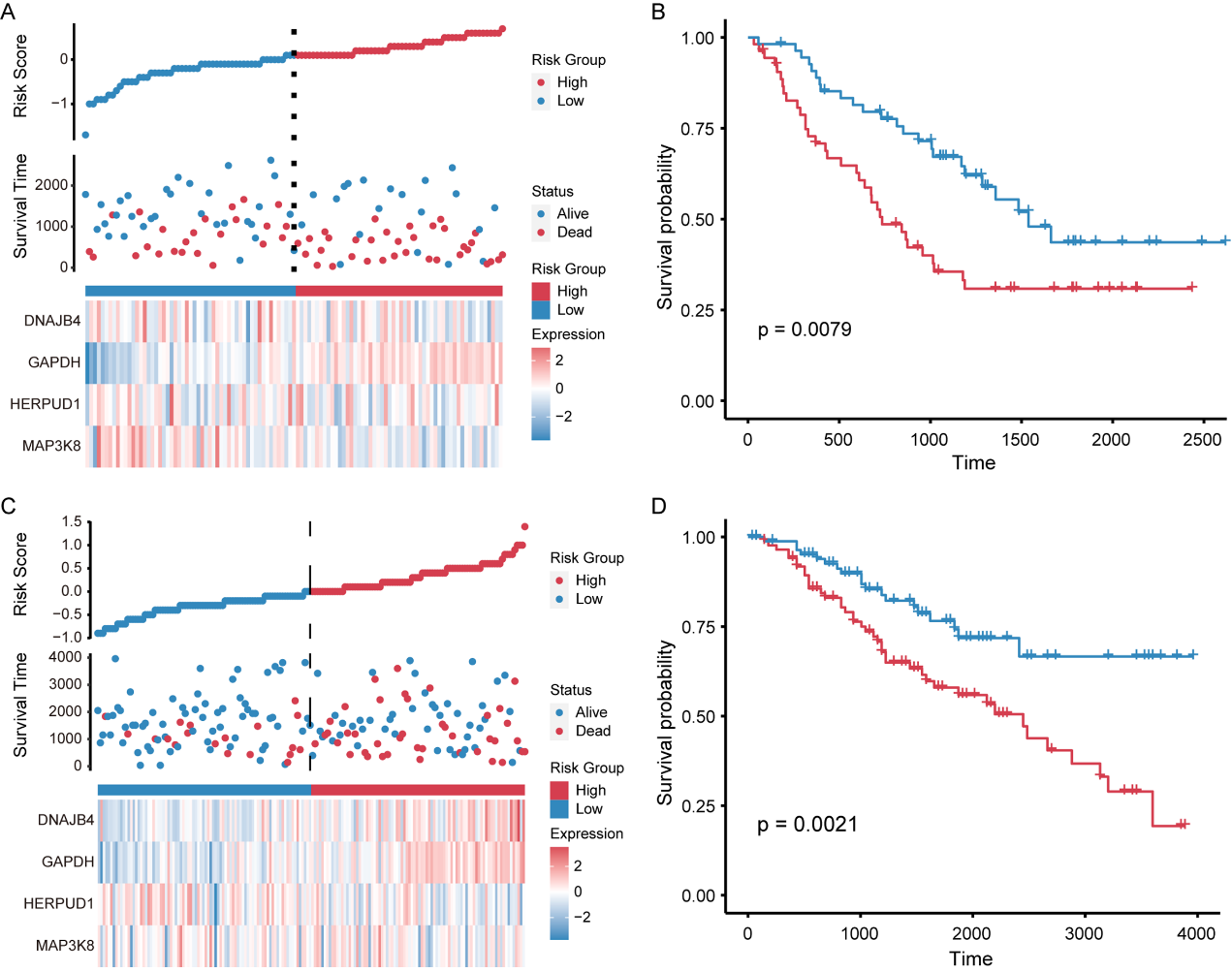


**Figure S6. (Related to Figure 5) (A)** This graph reflects the expression levels of four prognostic markers for GSE3141 series as well as the survival status and risk scores of the patients. **(B)** Kaplan-Meier (KM) curves for the survival time (days) of NSCLC samples from GES3141 series in high- and low-risk categories. The log-rank test was used to calculate statistical significance. **(C)** Same as in **(A)** but for GSE42127 series. **(D)** Same as in **(B)** but for the survival time (days) of NSCLC samples from GSE42127 series.

**Supplementary Tables**

**Table S1: The information of samples used in this study**

| Sequencing technology | Sample | Cell/sample number | Tissue | Sex | Diagnosis |
| --- | --- | --- | --- | --- | --- |
| Single-cell | GSM3304007 | 1832 | Tumor | Male | Lung adenocarcinoma |
|  | GSM3304009 | 1304 | Tumor | Female | Lung squamous cell carcinoma |
|  | GSM3304011 | 328 | Tumor | Female | Lung adenocarcinoma |
|  | GSM3304013 | 1423 | Tumor | Female | Lung adenocarcinoma |
| Bulk | TCGA-LUAD | 527 | Tumor | / | Lung adenocarcinoma |
|  | TCGA-LUSC | 503 | Tumor | / | Lung squamous cell carcinoma |
|  | GSE3141 | 111 | Tumor | / | Lung adenocarcinoma |
|  | GSE42127 | 176 | Tumor | / | Lung squamous cell carcinoma (43); Lung adenocarcinoma (133) |

**Table S2: Up-regulated metabolic pathways in each epithelial cell cluster**

| Patient | cluster | Metabolic pathway | Num |
| --- | --- | --- | --- |
| P1 | 0 | "Glycolysis / Gluconeogenesis", "Citrate cycle (TCA cycle)", "Pentose phosphate pathway", "Pentose and glucuronate interconversions", "Fructose and mannose metabolism", "Pyruvate metabolism", "Oxidative phosphorylation", "Fatty acid elongation", "Glycerophospholipid metabolism", "Ether lipid metabolism", "Arachidonic acid metabolism", "Biosynthesis of unsaturated fatty acids", "Purine metabolism", "Pyrimidine metabolism", "Cysteine and methionine metabolism", "Valine, leucine and isoleucine degradation", "beta-Alanine metabolism", "Glutathione metabolism", "Glycosaminoglycan degradation", "Riboflavin metabolism", "Pantothenate and CoA biosynthesis" | 21 |
|  | 1 | "Fatty acid biosynthesis", "Fatty acid degradation" | 2 |
| P2 | 0 | / | 0 |
|  | 1 | "Pentose and glucuronate interconversions", "Glyoxylate and dicarboxylate metabolism", "Propanoate metabolism", "Butanoate metabolism", "Sulfur metabolism", "Fatty acid biosynthesis", "Fatty acid degradation", "Sphingolipid metabolism", "Arachidonic acid metabolism", "Tryptophan metabolism", "Mucin type O-glycan biosynthesis", "Glycosaminoglycan biosynthesis - keratan sulfate", "Glycosphingolipid biosynthesis - ganglio series", "Nicotinate and nicotinamide metabolism", "Pantothenate and CoA biosynthesis", "Porphyrin and chlorophyll metabolism", "Metabolism of xenobiotics by cytochrome P450", "Drug metabolism - cytochrome P450" | 18 |
|  | 2 | "Glycolysis / Gluconeogenesis", "Citrate cycle (TCA cycle)", "Pentose phosphate pathway", "Starch and sucrose metabolism", "Amino sugar and nucleotide sugar metabolism", "Butanoate metabolism", "Purine metabolism", "Cysteine and methionine metabolism", "Valine, leucine and isoleucine degradation", "Glutathione metabolism", "Glycosaminoglycan biosynthesis - chondroitin sulfate / dermatan sulfate", "Glycosphingolipid biosynthesis - lacto and neolacto series", "Riboflavin metabolism", | 13 |
| P3 | 0 | "Glycolysis / Gluconeogenesis", "Pentose phosphate pathway", "Pentose and glucuronate interconversions", "Fructose and mannose metabolism", "Starch and sucrose metabolism", "Pyruvate metabolism", "Glyoxylate and dicarboxylate metabolism", "Oxidative phosphorylation", "Fatty acid biosynthesis", "Steroid biosynthesis", "Steroid hormone biosynthesis", "Glycerolipid metabolism", "Ether lipid metabolism", "Sphingolipid metabolism", "Arachidonic acid metabolism", "Purine metabolism", "Pyrimidine metabolism", "Alanine, aspartate and glutamate metabolism", "Glycine, serine and threonine metabolism", "Cysteine and methionine metabolism", "Lysine degradation", "Glutathione metabolism", "N-Glycan biosynthesis", "Mucin type O-glycan biosynthesis", "Other types of O-glycan biosynthesis", "Glycosaminoglycan biosynthesis - keratan sulfate", "Glycosylphosphatidylinositol (GPI)-anchor biosynthesis", "Glycosphingolipid biosynthesis - lacto and neolacto series", "Glycosphingolipid biosynthesis - globo and isoglobo series", "Glycosphingolipid biosynthesis - ganglio series", "Riboflavin metabolism", "Nicotinate and nicotinamide metabolism", "One carbon pool by folate", "Porphyrin and chlorophyll metabolism", "Terpenoid backbone biosynthesis", "Drug metabolism - other enzymes", | 36 |
| P4 | 0 | "Citrate cycle (TCA cycle)", "Pentose phosphate pathway", "Fructose and mannose metabolism", "Galactose metabolism", "Pyruvate metabolism", "Butanoate metabolism", "Fatty acid elongation", "Steroid biosynthesis", "Glycerolipid metabolism", "Glycerophospholipid metabolism", "Biosynthesis of unsaturated fatty acids", "Alanine, aspartate and glutamate metabolism", "Cysteine and methionine metabolism", "Arginine biosynthesis", "Histidine metabolism", "Glutathione metabolism", "Glycosylphosphatidylinositol (GPI)-anchor biosynthesis", "Glycosphingolipid biosynthesis - globo and isoglobo series", "Other glycan degradation", "Nicotinate and nicotinamide metabolism", "Porphyrin and chlorophyll metabolism", "Terpenoid backbone biosynthesis", | 22 |
|  | 1 | / | 0 |
|  | 2 | "Citrate cycle (TCA cycle)", "Pentose phosphate pathway", "Galactose metabolism", "Starch and sucrose metabolism", "Amino sugar and nucleotide sugar metabolism", "Pyruvate metabolism", "Glyoxylate and dicarboxylate metabolism", "Oxidative phosphorylation", "Fatty acid elongation", "Steroid biosynthesis", "Steroid hormone biosynthesis", "Glycerolipid metabolism", "Glycerophospholipid metabolism", "Ether lipid metabolism", "Sphingolipid metabolism", "Biosynthesis of unsaturated fatty acids", "Alanine, aspartate and glutamate metabolism", "Cysteine and methionine metabolism", "Lysine degradation", "Arginine biosynthesis", "Histidine metabolism", "Glutathione metabolism", "N-Glycan biosynthesis", "Glycosaminoglycan degradation", "Glycosylphosphatidylinositol (GPI)-anchor biosynthesis", "Nicotinate and nicotinamide metabolism", "Porphyrin and chlorophyll metabolism", "Metabolism of xenobiotics by cytochrome P450", "Drug metabolism - cytochrome P450", "Drug metabolism - other enzymes", | 30 |

**Table S3: The function of target genes positively related to the differentiation of CD4+ T cells**

| **Gene Symbol** | **Protein Function (Protein Atlas)** | **Hallmark Gene Sets** |
| --- | --- | --- |
| PPP1R15A | Predicted intracellular proteins; Cancer-related genes: Candidate cancer biomarkers | (M5896) HALLMARK TGF BETA SIGNALING; (M5890) HALLMARK TNFA SIGNALING VIA NFKB; (M5891) HALLMARK HYPOXIA |
| DUSP1 | Enzymes; Predicted intracellular proteins; ENZYME proteins: Hydrolases; Cancer-related genes: Candidate cancer biomarkers | (M5942) HALLMARK UV RESPONSE DN; (M5890) HALLMARK TNFA SIGNALING VIA NFKB; (M5891) HALLMARK HYPOXIA |
| PPP4C | Enzymes; Predicted intracellular proteins; ENZYME proteins: Hydrolases | (M5921) HALLMARK COMPLEMENT |
| FAS | CD markers; Disease related genes; Cancer-related genes: Candidate cancer biomarkers; Candidate cardiovascular disease genes; Predicted secreted proteins | (M5897) HALLMARK IL6 JAK STAT3 SIGNALING; (M5902) HALLMARK APOPTOSIS; (M5913) HALLMARK INTERFERON GAMMA RESPONSE |
| PMAIP1 | Predicted intracellular proteins | (M5902) HALLMARK APOPTOSIS; (M5906) HALLMARK ESTROGEN RESPONSE EARLY |
| TXNIP | Predicted intracellular proteins; Cancer-related genes: Mutational cancer driver genes | (M5911) HALLMARK INTERFERON ALPHA RESPONSE; (M5902) HALLMARK APOPTOSIS; (M5913) HALLMARK INTERFERON GAMMA RESPONSE |

**Table S4: The function of target genes positively related to the differentiation of CD8+ T cells**

| **Gene Symbol** | **Protein Function (Protein Atlas)** | **Hallmark Gene Sets** |
| --- | --- | --- |
| CCL5 | Cancer-related genes: Candidate cancer biomarkers; Predicted secreted proteins | (M5890) HALLMARK TNFA SIGNALING VIA NFKB; (M5913) HALLMARK INTERFERON GAMMA RESPONSE; (M5921) HALLMARK COMPLEMENT |
| IFNG | FDA approved drug targets: Small molecule drugs; Disease related genes; Cancer-related genes: Candidate cancer biomarkers; Predicted secreted proteins | (M5950) HALLMARK ALLOGRAFT REJECTION; (M5956) HALLMARK KRAS SIGNALING DN |
| HSPD1 | Predicted intracellular proteins; Potential drug targets; ENZYME proteins: Hydrolases; Enzymes; Disease related genes; Cancer-related genes: Candidate cancer biomarkers | (M5928) HALLMARK MYC TARGETS V2; (M5924) HALLMARK MTORC1 SIGNALING; (M5926) HALLMARK MYC TARGETS V1 |
